# Heats of Combustion and Formation of Trimethylborane, Triethylborane, and Tri-*n*-butylborane

**DOI:** 10.6028/jres.065A.025

**Published:** 1961-06-01

**Authors:** Walter H. Johnson, Marthada V. Kilday, Edward J. Prosen

## Abstract

The heats of combustion at 25 °C of trimethylborane (liquid), triethylborane (liquid), and tri-*n*-butylborane (liquid), to form crystalline boric acid, liquid water, and gaseous carbon dioxide have been determined to be −2989.4 ±22.4 kj/mole (−714.48 ±5.36 kcal/mole), −4975.6 ±15.1 kj/mole (−1189.2 ±3.6 kcal/mole), and −8901.0 ±10.2 kj/mole (−2127.4 ±2.4 kcal/mole), respectively. These data, combined with the heats of formation of boric acid, carbon dioxide, and water give −34.79 ±5.40, −47.2 ±3.7, and −83.2 ±2.5 kcal/mole at 25 °C for the standard heats of formation of trimethylborane, triethylborane, and tri-*n*-butylborane, respectively. The data of other investigators are discussed briefly.

## 1. Introduction

The alkyl boranes comprise a unique and rather extensive group of compounds somewhat analagous to the hydrocarbons. As in the case of the hydrocarbons, a knowledge of the thermochemistry of the lower members is necessary for calculating the thermodynamic properties of the other members of groups from bond energies and for determining the effects of molecular structure. The lower members of the group are extremely pyrophoric; trimethylborane and triethylborane ignite immediately upon exposure to air. Tri-*n*-butylborane also oxidizes rapidly in air but takes fire only when spread over a fairly large surface.

## 2. Materials

The samples of trimethylborane, triethylborane, and tri-*n*-butylborane were obtained from the Olin Mathieson Chemical Corporation. The trimethylborane was purified by means of fractional crystallization 1 and the purity was determined to be 99.9 mole percent from calorimetric freezing-point determinations.^2^ The material was stored in a stainless steel cylinder at −80 °C.

The purities of the triethylborane and tri-*n*-butylboranewere estimated to be about 99 percent from the quantities of carbon dioxide produced in the combustions. The samples were prepared by the reaction of the appropriate alkyl magnesium bromide with boron trifluoride. The most likely impurities were the mono- and di-alkyl substitution products. Therefore, the quantity of carbon dioxide found in the combustion products was assumed to be an indication of the purity of the sample. No attempts were made to purify these materials further because of the small quantities of material available.

The oxygen was purified by passing it through an oxidizer (containing copper oxide at 600 °C) and through an absorber containing Ascarite.

## 3. Apparatus

The calorimeter, the thermometric system, and the general calorimetric procedure have been described previously [1, 2, 3].^3^ A twin-valve stainless-steel bomb, fitted with a gold gasket, was used for the combustion experiments on trimethylborane; the volume of the bomb was 376 ml. A similar bomb, with a Teflon gasket and having a volume of 385 ml, was used for the experiments on triethylborane and on tri-*n*-butylborane. The apparatus used for determination of the quantity of carbon dioxide has been described [2].

## 4. Procedure

### 4.1. Trimethylborane

Spherical Pyrex-glass bulbs, having capacities of 0.5 to 1.0 ml, were sealed to standard taper joints, weighed, and attached to a distillation manifold with wax (Apiezon “W”). Samples of trimethylborane, weighing 0.2 to 0.4 g, were distilled into the bulbs which were then sealed in vacuo, warmed to room temperature, and weighed. The bulbs were then stored at −80 °C.

The bulb containing the sample was wrapped with glass wool, placed in a platinum crucible and weighed. About 0.1 g of *n*-hexadecane (NBS Standard Sample 568) was dropped on the glass wool and the quantity determined by re-weighing the crucible. A platinum wire fuse, 0.076 mm in diameter and 50 mm long, was connected between the bomb electrodes so that it was located slightly above and near the side of the crucible. A weighed strip of filter paper was then attached to the fuse and placed so that it extended to the bottom of the crucible.

Two milliliters of water were placed in the bomb which was then closed and placed in the calorimeter can with enough water to cover the surface of the bomb. The bomb was then flushed with purified oxygen (to remove nitrogen) and filled with oxygen to a pressure of 30 atm at 25 °C. The can, together with the water and the charged bomb, was weighed and the quantity of water adjusted to 1,000.0 g less than the total amount required for the “standard” calorimeter system. The can with its contents was then transferred to the calorimeter and 1,000 g of water was added from a weighed flask. After the transfer of water the empty flask was re-weighed to determine accurately the amount of water added.

It was necessary to keep the bomb submerged during and after filling to avoid the danger of an accident if the bulb broke under the oxygen pressure. Because of the difficulty of manipulating the completely filled calorimeter can, the 1,000 g portion of water was added after the can was inserted into the calorimeter. Because the quantity of water was not exactly reproduced from experiment to experiment, a small but accurately determined correction for the resulting deviation in the heat capacity of the calorimeter system was made in each case.

The gaseous contents of the bomb were analyzed after each experiment to determine the quantity of carbon dioxide [2]. In the trimethylborane experiments, the liquid and solid materials remaining in the bomb were washed with hot water into a boron-free beaker. The resulting solution was filtered through a sintered-glass funnel to remove the glass fragments and the insoluble residue. The filtrate was titrated for nitric acid, and for boric acid in the presence of mannitol, using standard alkali solution; the end points of the titrations were determined by means of a Beckman *p*H-meter.

The solid portion collected in the filter was digested with nitric acid to dissolve elemental boron; analysis of the resulting solution showed no measurable quantity of boric acid, although the solution was so highly buffered that precise measurements were not possible. Portions of the insoluble residue were found by X-ray diffraction studies 4 to contain traces of B_4_C, the only crystalline material identified.

### 4.2. Triethylborane and Tri-*n*-butylborane

The samples were placed in spherical soft-glass bulbs by means of a hypodermic syringe; this operation was performed in a dry-box containing an argon atmosphere. Rubber caps were placed over the tips of the bulbs which were then removed from the dry-box, cooled in an ice-bath, and sealed.

The bulb containing the sample was placed in a platinum crucible. An iron wire fuse, 50 mm long and 0.127 mm in diameter, was connected between the electrodes and adjusted to be about 1 mm directly above the sample bulb. One milliliter of water was placed in the bomb which was then closed. The flushing, filling and transfer operations, and the subsequent analyses of the gaseous combustion products were the same as described for the experiments on trimethylborane.

### 4.3. Calibration

The calorimetric systems were calibrated by means of a series of combustion experiments on benzoic acid, NBS Standard Sample 39 g. The certified value for the heat of combustion of this sample in the standard bomb process is 26433.8 ±2.2 j/g at 25 °C. This value, when corrected for deviations from the standard bomb conditions, gave 26430.9 j/g at 28 °C [4]. The energy equivalent of the calorimetric system used for the experiments on trimethylborane was determined to be 141754.7 ±7.0 j/ohm.

The energy equivalent of the calorimetric system used for the experiments on triethylborane and tri-*n*-butylborane differed significantly from that used with trimethylborane because of the substitution of a larger bomb. The certified value for the heat of combustion of benzoic acid in the bomb process, corrected for deviations from the standard bomb process, was determined to be 26427.1 j/g at 29.8 °C. The energy equivalent of the calorimeter system was determined to be 136080.6 j/ohm.

## 5. Units and Conversion Factors

All atomic weights were taken from the 1957 International Table of Atomic Weights [5]. All weighings were corrected to weight in a vacuum.

The unit of energy was the joule; for conversion to the conventional thermo chemical calorie, one calorie was taken as 4.1840 joules.

## 6. Results and Calculations

The heat of combustion of *n*-hexadecane was taken as −47155.2 ±7.7 j/g for the bomb process at 28 °C [6]. The heat of combustion of the filter paper was determined, in a separate series of experiments, to be 17084.5 ±8.0 j/g for the bomb process at 28 °C; analyses of the combustion products gave 1.599 g CO_2_/g. The density of trimethylborane (liquid) at 25 °C was taken as 0.625 g/cm^3^ [7]; the heat capacity for the liquid at 25 °C was estimated as 30.8 cal/mole °C [8]. The densities of triethylborane and of tri-*n*-butylborane were taken as 0.74 and 0.68 g/cm^3^ respectively from the volumes and weights of the sample bulbs; the heat capacity of triethylborane was taken as 57.6 cal/mole °C [8], and the heat capacity of tri-*n*-butylborane was estimated to be 107 cal/mole °C.

The results of the experiments on the combustion of trimethylborane are given in table 1 where Δ*Rc* is the corrected temperature rise of the calorimetric system [4], −Δ*e* is the deviation in the energy equivalent of the actual calorimetric system from that of the calibrated system, *q_N_* is a correction for the heat of formation of nitric acid [4], WC is the Washburn correction [4], *q_B_*_(am)_ is the correction for converting amorphous boron to crystalline boric acid, and Δ*E°* is the heat of the constant-volume process at 28 °C with all reactants and products in their respective thermodynamic standard states. The Washburn correction has been modified for these calculations to include the heat of solution of the combustion products in the aqueous portion. The mass of *n*-C_16_H_34_, of CO_2_, and of sample, S, given in table 1 are corrected to vacuum; the weight of filter paper is not corrected to vacuum. Boron in the sample which was not found as boric acid was assumed to be present in the insoluble products as amorphous boron. For the conversion of amorphous boron, gaseous oxygen, and liquid water to crystalline boric acid, we used −670 kj/mole of amorphous boron as the heat of reaction. The calculation of Δ*E°* was based on the moles of trimethylborane calculated from the sample weight, because the use of auxiliary materials introduced errors in the determination of carbon dioxide formed in the combustion of the sample. The average ratio of the mass of carbon dioxide found in the products of combustion to the mass of carbon dioxide calculated from the masses of sample and *n*-hexadecane, and weight of filter paper was 0.9943 where the standard deviation of the mean was ±0.0024.

The results of the experiments with triethylborane and with tri-*n*-butylborane are given in tables 2 and 3 respectively. In each case the calculations were based on the mass of carbon dioxide produced in the combustion process.

The results, corrected to the constant-pressure process at 25 °C, correspond to the reactions:

$$\begin{array}{l} B{\left({\text{CH}}_{3}\right)}_{3}\left(\text{liq}\right)+6\;O_{2}\left(g\right)\to \\ \;H_{3}{\text{BO}}_{3}\left(c\right)+3\;{\text{CO}}_{2}\left(g\right)+3H_{2}O\left(\text{liq}\right),\\ \;\Delta H{}^{\circ}\left(25\;{}^{\circ}C\right)=-2989.4\pm 22.4\;\text{kj}/\text{mole}\\ \;=-714.48\pm 5.36\;\text{kcal}/\text{mole}, \end{array}$$ (1)

$$\begin{array}{l} B{\left(C_{2}H_{5}\right)}_{3}\left(\text{liq}\right)+21/2\;O_{2}\left(g\right)\to \\ \;H_{3}{\text{BO}}_{3}\left(c\right)+6\;{\text{CO}}_{2}\left(g\right)+6H_{2}O\left(\text{liq}\right),\\ \;\Delta H{}^{\circ}\left(25\;{}^{\circ}C\right)=-4975.6\pm 15.1\;\text{kj}/\text{mole}\\ \;=-1189.2\pm 3.6\;\text{kcal}/\text{mole}, \end{array}$$ (2)

$$\begin{array}{l} B{\left(n{\text{-C}}_{4}H_{9}\right)}_{3}\left(\text{liq}\right)+39/2\;O_{2}\left(g\right)\to \\ \;H_{3}{\text{BO}}_{3}\left(c\right)+12\;{\text{CO}}_{2}\left(g\right)+12H_{2}O\left(\text{liq}\right),\\ \;\Delta H{}^{\circ}\left(25\;{}^{\circ}C\right)=-8901.0\pm 10.2\;\text{kj}/\text{mole}\\ \;=-2127.4\pm 2.4\;\text{kcal}/\text{mole}. \end{array}$$ (3)

The uncertainties assigned to the above values have been taken as twice the standard deviation (or twice the average deviation) of the mean of the experimental values, combined with reasonable estimates of all other known sources of error.

The heats of formation of carbon dioxide (gas) and of water (liquid) were taken from the literature [9]; the heat of formation of crystalline boric acid was taken as −262.16±0.32 kcal/mole [10]. These were combined with the values given above to obtain the heats of formation of the liquids given in table 4.

The heat of vaporization of trimethylborane at 25 °C was calculated to be 4.83 kcal/mole from the calorimetric data of Furukawa and Park [8] and the vapor pressure data reported by Furukawa and Park [8] and by Stock and Zeidler [7]. The heat of vaporization of triethylborane at 25 °C was calculated to be 8.8 kcal/mole from the vapor pressure data of Furukawa and Park [8]. The heat of vaporization of tri-*n*-butylborane was calculated to be 13.8 kcal/mole at 25 °C from the vapor pressure equation reported by Skinner and Tees [11]. These values were used to obtain the heats of formation in the gas phase for trimethylborane, triethylborane, and tri-*n*-butylborane given in table 4.

These heats of formation have been combined with the data on the hydrocarbons [12] to obtain values for the average B—C bond energy. The heats of formation at 25 °C of B(g), H(g), and C(g) were taken as 135.2, 52.1, and 171.3 kcal/g-atom, respectively [13]. The heat of formation of methane was used to obtain the average bond energy of the C—H bond in the methyl group. Combination of this value with the heat of formation of trimethylborane leads to 84.4 kcal/mole for the B—C bond. The data for ethane, propane, and butane were used to obtain values of the average bond energies of the C—H and C—C bonds in the higher alkyl groups. Combination of these values with the heats of formation of triethylborane and tri-*n*-butylborane leads to values of 84.3 and 84.3 kcal/mole, respectively, for the B—C bond. This value, together with the values of the C—C and C—H bond energies obtained for C_2_H_6_, C_3_H_8_, and *n*-C_4_H_10_, was used to calculate the heat of formation of tri-*n*-propylborane given in table 4.

The values for the isomeric propyl and butyl compounds given in table 4 were obtained from a comparison with the data on hydrocarbons [12]. The values, except for tri-*t*-butylborane, include only the effects of isomerization in the alkyl groups. The value for tr-*n*-butylborane includes a repulsive energy of 1 kcal for interaction between each pair of groups. It is felt that the uncertainty of these calculated values is ±5 kcal/mole.

Long and Norrish [14] have also measured the heat of combustion of liquid trimethylborane, but under slightly different conditions. A correction of their data for the value taken for the heat of combustion of the benzoic acid with which they calibrated their calorimetric system, for the heat of solution of the boric acid formed, for the atomic weight of carbon, and to 25 °C yields for the liquid: Δ*Hc°* = −714.80 kcal/mole and Δ*Hf°* = −34.47 kcal/mole.

Tannenbaum and Schaeffer [15] measured the heat of combustion of tri-*n*-butylborane and obtained for the reaction:

$$\begin{array}{l} B{\left(n{\text{-C}}_{4}H_{9}\right)}_{3}\left(\text{liq}\right)+39/2\;O_{2}\left(g\right)\to 1/2B_{2}O_{3}\left(c\right)\\ \;+12{\text{CO}}_{2}\left(g\right)+27H_{2}O\left(\text{liq}\right)\\ \;\Delta H{}^{\circ}\left(25\;{}^{\circ}C\right)=-2117.6\;\text{kcal}/\text{mole}. \end{array}$$

This value yields −2124.6 kcal/mole for the process corresponding to equation 3.

The data reported by Long and Norrish and by Tannenbaum and Schaeffer are in excellent agreement with the results of this investigation.

## 7. Discussion

Measurements of the heats of combustion of the alkylboranes, as well as of boron compounds in general, are subject to several uncertainties regarding the nature and thermodynamic states of the combustion products. The major portion of the boron is burned to form boric oxide which is then hydrated to boric acid; it is not known, however, if the hydration process is completed during the period when calorimeter temperatures are being observed. There is also some uncertainty regarding the amount and the concentration of aqueous boric acid. Although the assumption was made that the liquid water in the bomb was saturated with respect to boric acid at the final calorimeter temperature, this is very probably not true. Later we plan to do more work to supplement the two experiments reported for triethylborane and for tri-*n*-butylboran, and we hope at that time to obtain more information regarding the final state of the products.

It was necessary to use rigid Pyrex bulbs for the trimethylborane samples because of the high vapor pressure of the compound and to permit transfer of the material in vacuo. The use of flexible soft-glass bulbs for the triethylborane and tri-*n*-butylborane samples was attempted but they invariably broke during filling of the bomb; it appears that these liquids have abnormally high compressibility coefficients.

The use of soft glass for containing the sample is generally preferred, except in those cases which require the sample to be frozen or cooled to very low temperatures, because the bulbs are usually fused into small globules during the combustion process. Rigid Pyrex bulbs are usually not fused and some method must be employed to shatter the bulb completely; the sample may otherwise be entrapped within the bulb and burn in a limited supply of oxygen.

In the case of trimethylborane, the residues contained small amounts of a dark material, which was not soluble in water, and which indicated the presence of elemental carbon, elemental boron, or boron carbide. Although the amounts of elemental boron were too small for determination by the analytical methods employed, the possibility of its presence could not be eliminated. The quantity of boron carbide, although detected in the residue, was assumed to be negligible.

The residues from the triethylborane combustions appeared to contain extremely small amounts of water-insoluble materials; the residues from the tri-*n*-butylborane contained no visible traces of insoluble material. This trend was to be expected, however, since the quantities of elemental boron or of boron carbide would be a function of the total amount of boron in the sample.

The heats of combustion and formation given in this paper are believed to be accurate within the limits of the assigned uncertainties. Additional measurements on compounds of higher purity would not appreciably reduce these uncertainties because they are chiefly caused by our inability at the present time to characterize accurately the nature and thermodynamic states of the combustion products.

## Figures and Tables

**Table 1:** Results of the experiments on the combustion of trimethylborane

Experiment	Δ*Rc*	Δ*e*	*n*-C_16_H_34_	Filter paper	CO_2_	H_3_BO_3_ titr.	*q*_*N*_	WC	*q*_*B*(am)_	S	−Δ*E*° (28 °C)
											
	*Ohm*	*j/ohm*	*g*	*g*	*g*	*Mole*	*j*	*j*	*j*	*Mole*	*kj/mole*
1	0.144332	72.5	0.12816	0.01085	1.04646	0.004726	0.5	3.0	40	0.0047855	2983.6
2	.125478	41.2	.13063	.01145	0.92999	.003657	1.0	2.6	174	.0039160	2964.0
3	.116465	24.0	.14520	.01136	.88888	.003053	0.4	2.4	113	.0032211	2974.4
4	.156235	68.8	.09554	.01185	1.08877	.005668	.0	3.2	129	.0058611	2998.8
5	.127848	34.4	.14098	.01030	0.95582	.003633	.0	2.6	107	.0037929	3007.7
6	.183669	38.7	.10816	.00837	1.26985	.006805	.2	3.0	190	.0070885	2960.6
	
Mean											2981.5
Standard deviation of the mean											±7.7

**Table 2:** Results of the experiments on the combustion of triethulborane

Experiment	Δ*Rc*	Δ*e*	*q*_*ign*_	*q*_*N*_	WC	CO_2_	−Δ*E°* (29.8 °C)
							
	*Ohm*	*j*/*ohm*	*j*	*j*	*j*	*g*	*kj*/*g* CO_2_
1	0.062858	−23.8	33.9	0.6	0.4	0.45323	18.793
2	.102274	1.6	34.0	1.0	.9	.73851	18.797
	
Mean							18.795

**Table 3:** Results of the experiments on the combustion of tri-n-butylborane.

Experiment	Δ*Rc*	Δ*e*	*q*_*ign*_	*q*_*N*_	WC	CO_2_	−Δ*E°* (29.8 °C)
							
	*Ohm*	*j*/*ohm*	*j*	*j*	*j*	*g*	*kj*/*g* CO_2_
1	0.123530	−20.8	33.4	1.0	2.0	0.99797	16.805
2	.101518	1.6	33.1	1.0	1.6	.81913	16.822
	
Mean							16.814

**Table 4:** Heats of formation of trialkylboranes ΔHf° (25 °C) in kcal/mole

	Liquid	Gas
		
(CH_3_)_3_B	−34.79	−29.96
(C_2_H_5_)_3_B	−47.2	−38.4
(*n*-C_3_H_7_)_3_B		−52.3
(*i*-C_3_H_7_)_3_B		−57.4
(*n*-C_4_H_9_)_3_B	−83.2	−69.4
(*i*-C_4_H_9_)_3_B		−74.5
(*sec*-C_4_H_9_)_3_B		−72.6
(*t*-C_4_H_9_)_3_B		−85.6

## Footnotes

1 Pure Substances Section of the Chemistry Division.

2 Thermodynamics Section of the Heat Division.

3 Figures in brackets indicate literature references at the end of this paper.

4 Constitution and Microstructure Section of the Mineral Products Division.
